# The comparison of Canopeo and samplepoint for measurement of green canopy cover for forage crops in India

**DOI:** 10.1016/j.mex.2022.101916

**Published:** 2022-11-09

**Authors:** Prabhu Govindasamy, Sonu Kumar Mahawer, Debalin Sarangi, Hanamant M. Halli, T.K. Das, Rishi Raj, Vijay Pooniya, L. Muralikrishnan, Sunil Kumar, Amaresh Chandra

**Affiliations:** a Divison of Crop Production, ICAR-Indian Grassland and Fodder Research Institute, Jhansi, India; b Division of Agronomy, ICAR-Indian Agricultural Research Institute, New Delhi, India; c Department of Plant Sciences, University of Wyoming, Laramie, WY, USA; d Divison of Seed Technology, ICAR-Indian Grassland and Fodder Research Institute, Jhansi, India; e School of Soil Stress Management, National Institute of Abiotic Stress Management, Pune, India; f Division of Agricultural Extension, ICAR-Indian Agricultural Research Institute, New Delhi, India; g Director, ICAR-Indian Grassland and Fodder Research Institute, Jhansi, India

**Keywords:** Crop cover, Non-destructive method, Nadir image, Photogrammetry

## Abstract

Canopy covers can be measured using destructive (visual) and non-destructive methods (spectral indices, photogrammetry, visual assessment, and quantum sensor). The precision of crop cover estimation, however, is dependent on the selection of appropriate methods. Studies were conducted at the Indian Grassland and Fodder Research Institute, Jhansi to compare the forage crops canopy cover estimated using photogrammetry software (Canopeo and SamplePoint) and visual assessments. Assessments were performed in three summer crops (corn, cowpea, and sorghum), two winter crops (Egyptian clover, and oats), and bare ground condition. For each plot, three nadir images (directly above the canopy) were captured using digital cameras from a height of 1.5 m above the soil surface between 10 AM to 2 PM on bright sunny days. The results indicated that the relationships between visual assessment and Canopeo (regression coefficient, (R^2^ = 0.96), visual assessment and SamplePoint (0.96), and Canopeo and SamplePoint (0.98) were linear when data were pooled across all the crops. SamplePoint and Canopeo is further, appropriate for cowpea (Pearson coefficient (*R* = 0.99 and 0.94), oats (0.92 and 0.97), and sorghum (0.46 and 0.51), respectively. SamplePoint and Canopeo are not suitable for berseem (-0.15) and corn (-0.61), respectively, due to dead residues after the first harvest in berseem and taller corn might have influenced the image quality. Therefore, the stage of the crop, the height of the crop, and dead residues around the plants can greatly influence the estimation of crop cover. In conclusion, the results indicated that this photogrammetry software can be used for non-destructive crop canopy measurement with the above-mentioned precautions in the forage crops tested.

• Forage canopy cover is estimated generally by visual scoring, and the outcome varies widely from person to person.
• Photogrammetry methods (Canopeo and SamplePoint) were positiviely correlated with visual scoring for cowpea, oats, and sorghum.
• However, Canopeo and SamplePoint may not suitable for taller crops like corn and ratoon crops like berseem.

## Graphical abstract

**Figure:**
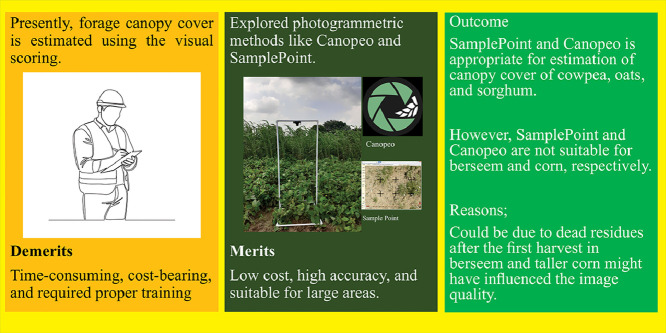

Specifications Table

**Table:** 

Subject area:	Agricultural and biological sciences
More specific subject area:	Agronomy
Name of your method:	Canopeo and SamplePoint
Name and reference of original method:	A. Patrignani, T. E. Ochsner, Canopeo: A powerful new tool for measuring fractional green canopy cover. Agron. J. 107 (2015) 2312–2320.
Resource availability:	Not applicable

## Background

Assessment of canopy cover is important to estimate the biomass and grain yield of a crop, as the photosynthetic activity of a crop and canopy cover (green areas) are correlated [1]. Common methods for measuring green canopy cover are visual assessments and using line quantum sensors [2,3]. However, they are time-consuming, cost-bearing, and required proper training. Digital image analysis software such as Canopeo and SamplePoint [4] are considered user-friendly and cost-effective methods for rapid and non-destructive measurement of canopy covers [5]. Photogrammetry technology, the science of measuring physical objects through photographic images and visual monitoring systems, is used for canopy measurement using Canopeo and SamplePoint. Moreover, the advancement and availability of photogrammetric software are helping to make a progress in input use efficiency, high throughput phenotyping, plant growth measurement, and plant health detection [6].

The photogrammetric software is quicker compared to the current methods used for green canopy assessment. A study conducted by Purcell [7] reported that the digital imagery method took 1 min 30s for image recording and determination of canopy cover in soybean (*Glycine max* L.), and that was comparable to the time required for a line quantum sensor data processor. For sorghum biomass estimation, digital image analysis using Canopeo was more efficient than visual methods [8]. A linear relationship was also found between Canopeo and line quantum sensor measurements for canopy cover in Soybean [9], which indicates that the digital imagery method or photogrammetry software are as efficient as the traditional method for canopy cover measurements. Additionally, Purcell^7^ noted that the influence of environmental factors such as soil color, soil moisture content, and the light intensity on canopy cover estimation using photogrammetry software are negligible.

SamplePoint is mostly used to identify the vegetative community, plant cover, status of chlorophyll and nitrogen content in plants [4,10,11,12]. Similar to Canopeo, SamplePoint is relatively a cost-effective technology to measure ground cover compared to the existing technologies such as visual assessment and can easily detect the changes in the ecological indicators [13]. Moreover, Nielsen et al. [14] mentioned that SamplePoint software took < 2 min to process 64 points in an image. SamplePoint was proven to be efficient to estimate residue cover of flax (*Linum usitatissimum* L.), oats (*Avena sativa* L.), pea (*Pisum sativum* ssp. *arvense* L. Poir), rapeseed (*Brassica napus* L.) and a mixture of 10 cover crop species in Nebraska, U.S.A. [14]. Similarly, Prabhakara et al. [15] also reported that SamplePoint was an efficient and user-friendly software to determine the proportion of dark and bright crop residue, light and dark bare soils, and green and yellow vegetation.

The photogrammetry tools such as Canopeo and SamplePoint are not widely used for forage research in India and no data is available on these software when compared with visual assessments for canopy measurement in forage crops. The objectives of this study were to (1) assess the performance of Canopeo and SamplePoint for canopy cover measurement in fodder corn (*Zea mays* L.), cowpea [*Vigna unguiculata* (L.) Walp], Egyptian clover (*Trifolium alexandrinum* L.), oats (*Avena sativa* L.), and sorghum [*Sorghum bicolor* (L.) Moench], and (2) compare the Canopeo and SamplePoint data for the canopy cover estimation with visual assessments.

### Software description

Canopeo, a digital image-based software for canopy measurement, is developed by Oklahoma State University and this application is available for Android and iOS devices with no costs (http://www.canopeoapp.com). It is an automatic color threshold (ACT) deduction software and measures the green ground cover based on red to green (R/G), blue to green (B/G) and an overload green index (2G – R – B) [5]. Canopeo, estimates green crop cover by turning green cover to white, and bare land area to black pixels, thereby it produces the percent white-colored pixels in a given image [16]. Furthermore, the pixel categorization using red to green and blue-to-green ratios in Canopeo is proven as an effective method to separate green surface with non-green surface. SamplePoint is also a no-cost computer-based software (http://www.samplepoint.org) developed by Booth et al. [4]. It works based on manual pixel classification (MPC) and targets up to 225 crosshairs per image. This software can distinguish broadleaf, grasses, shrub, cactus, litter, soil, stone, rock, and unknown by manual pixel classification which is an added advantage of this software compared to Canopeo. Based on the percentage of manual pixels for green canopy cover, soil, stone, and rocks, it provides the percentage of crop cover per unit area. While analysing the images the SamplePoint software overlay 100 crosshairs in each image and classify the objects in the image based on manual identification. Depending on the number of crosshairs intersecting green canopy out of 100 crosshairs counts is used to calculate the green cover percentage in a particular area.

### Forage crops

Corn (var., African tall), cowpea (var., Bundel Lobia 1), and sorghum (var., MP Chari) were planted in June immediately after the onset of the rainy season in Jhansi, India. The seeds were planted at the rate of 45, 40 and 35, kg ha^–1^ for corn, cowpea, and sorghum, respectively. For corn, a 40-cm row-spacing was used; however, 30-cm spacing was used for cowpea and sorghum. Winter crops including Egyptian clover (Bundel berseem 2) and oats (var., JHO 822) were planted in November at the seed rate of 25 and 90 kg ha^–1^, respectively. Fertilizers were applied following the recommended practices and soil test values. The research was repeated in 2018 and 2019 under the similar management practices at the Indian Grassland and Fodder Research Institute (25°29’48.4” N, 78°33’35.6” E, elevation 233 m) at Jhansi, India.

### Image capturing and processing

**Fig. 1:**
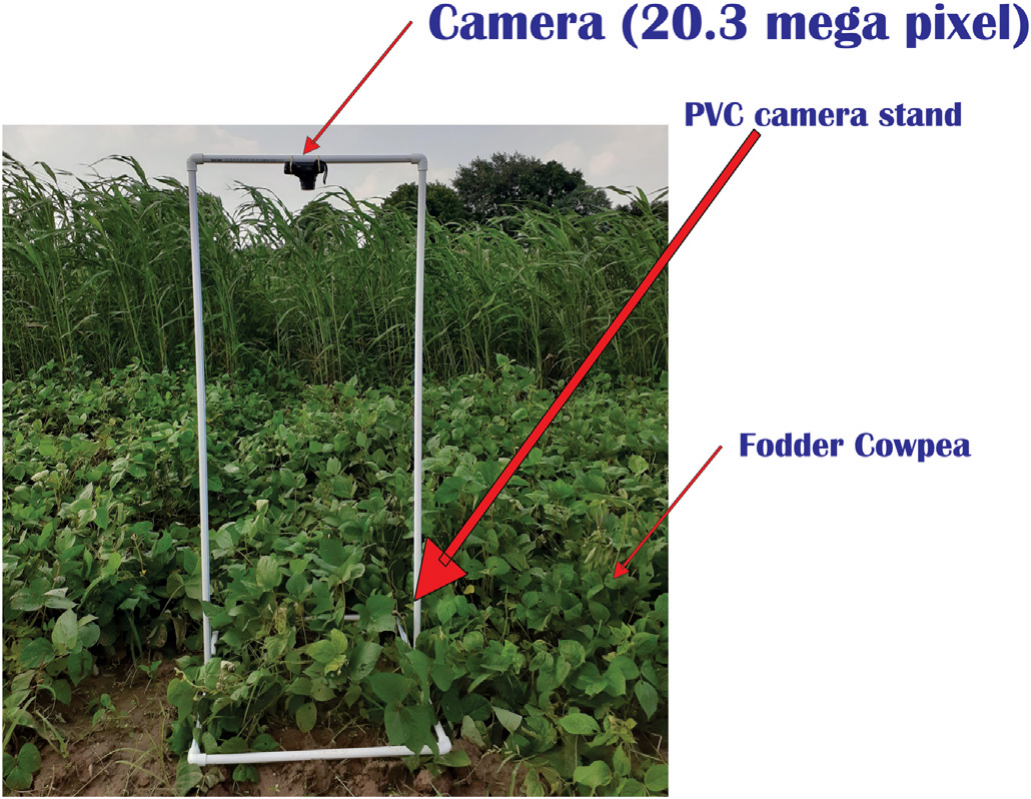
Stand built using PVC pipes to mount a camera for taking pictures in the experimental plots.

The images were captured in each research plots (corn, sorghum, cowpea, oats and berseem) using Canon SX710 HS (Canon Inc., Vienna, Austria) digital cameras (resolution 32 megapixels) between 10 AM to 2 PM on a bright sunny. Quality images are very important for assessing the quality of the crop environment and it is affected by distortion of clouds, rain, and frost, therefore cloud-free day (10 AM to 2 PM) is preferred for capturing images [17,18]. Camera resolution also plays an important role in getting quality images thus at least a 20-megapixel camera must be selected for this kind of photogrammetric study [19]. At the time of image capturing the age of crops was 45 days old. The nadir images (90° down-facing) of 0.5 m^2^ ground area were randomly taken at a height of 1.5 m from the crop canopy using a camera stand made using PVC pipes (Fig. 1). The nadir images are important for all the aerial imaging studies because they are effective in covering the surface of plants and flexible for restructuring [20]. To avoid the overestimation in measurement, appropriate precautions such as the distance between camera and crop, nadir imaging, ratio of red to green and blue to green were taken [21]. Three pictures were taken randomly from each plot for analysis in Canopeo and SamplePoint. Visual assessments of canopy cover at the time of image recording were performed by four people (to induce variability) at the same time as digital image recording and the Canopeo and SamplePoint data were compared with the visual assessment data for precision. Visual assessment is a traditional technique where humans visually estimate crop cover by eliminating the ground area. The procedures described by Patrignani and Ochsner [5] for Canopeo and Booth et al. [4] for SamplePoint were used and the details of Canopeo and SamplePoint software settings and plant characteristics were listed in Table 1. Images of JPEG format were used in this research.

**Table 1:** Software settings and plant characteristics at the time of image recording and processing.

Methods	Settings	Corn	Cowpea	Egyptian clover	Oats	Sorghum
Canopeo	R/G*	0.97	0.97	0.97	0.97	0.97
	B/G*	0.97	0.97	0.97	0.97	0.97
	Noise reduction	1	1	1	1	1
	Speed of processing per image	1.5 to 2 min	1.5 to 2 min	1.5 to 2 min	1.5 to 2 min	1.5 to 2 min
SamplePoint	Number of pixels	100	100	100	100	100
	Speed of processing per image	1 min	1 min	1 min	1 min	1 min
Visual assessment	Number of assessments	4	4	4	4	4
	Speed of assessment per image	> 2 min	> 2 min	> 2 min	> 2 min	> 2 min
Average plant height (cm)		85	48	42	45	75
Average Leaf width (cm)		6.5	8	1.45	1.8	3

⁎ R/G; Red and Green ratio and B/G; Blue and Green ratio

### Statistical analysis

Regression analysis was performed on the percent canopy cover (green) data obtained from Canopeo, SamplePoint, and visual assessments using the Proc REG procedure in SAS (version 9.3, SAS Institute Inc, Cary, NC). The significance of the slopes were determined at P = 0.05. Visual assessment method used as a check in this study, therefore we took average of four persons (replication) observation and used it for further analysis. Pearson correlation analyses were performed to check the relationship among Canopeo, SamplePoint, and visual assessments of canopy covers in individual crops using the Proc CORR procedure in Statistical Analytical System (SAS Institute Inc, Cary, NC). Regression plots were made in Sigma Plot (version 10, Systat Software, Inc., San Jose, California, USA). For both regression and correlation analysis, the percent canopy estimation of individual methods was used as a variable, for example in Fig. 5A, the percent canopy estimation of the visual assessment method was used as an X variable, and the percent canopy estimation of Canopeo as a Y variable. Likewise, for correlation analysis, the percent canopy estimation of the visual assessment method was used as variable 1, and the percent canopy estimation of Canopeo as variable 2 (Table 2).

**Table 2:** Correlation among visual assessment, Canopeo and SamplePoint values for different crops.

Crops	Visual vs. Canopeo	Visual vs. SamplePoint	Canopeo vs. SamplePoint
	Pearson correlations coefficients (R)		

Corn	-0.61	0.96	-0.38
Cowpea	0.94	0.99	0.97
Egyptian clover	0.95	-0.15	-0.44
Oats	0.97	0.92	0.98
Sorghum	0.51	0.46	0.99*

⁎ Data significance at *p*–value < 0.05

### Precision of Canopeo and SamplePoint on canopy cover estimation

**Fig. 2:**
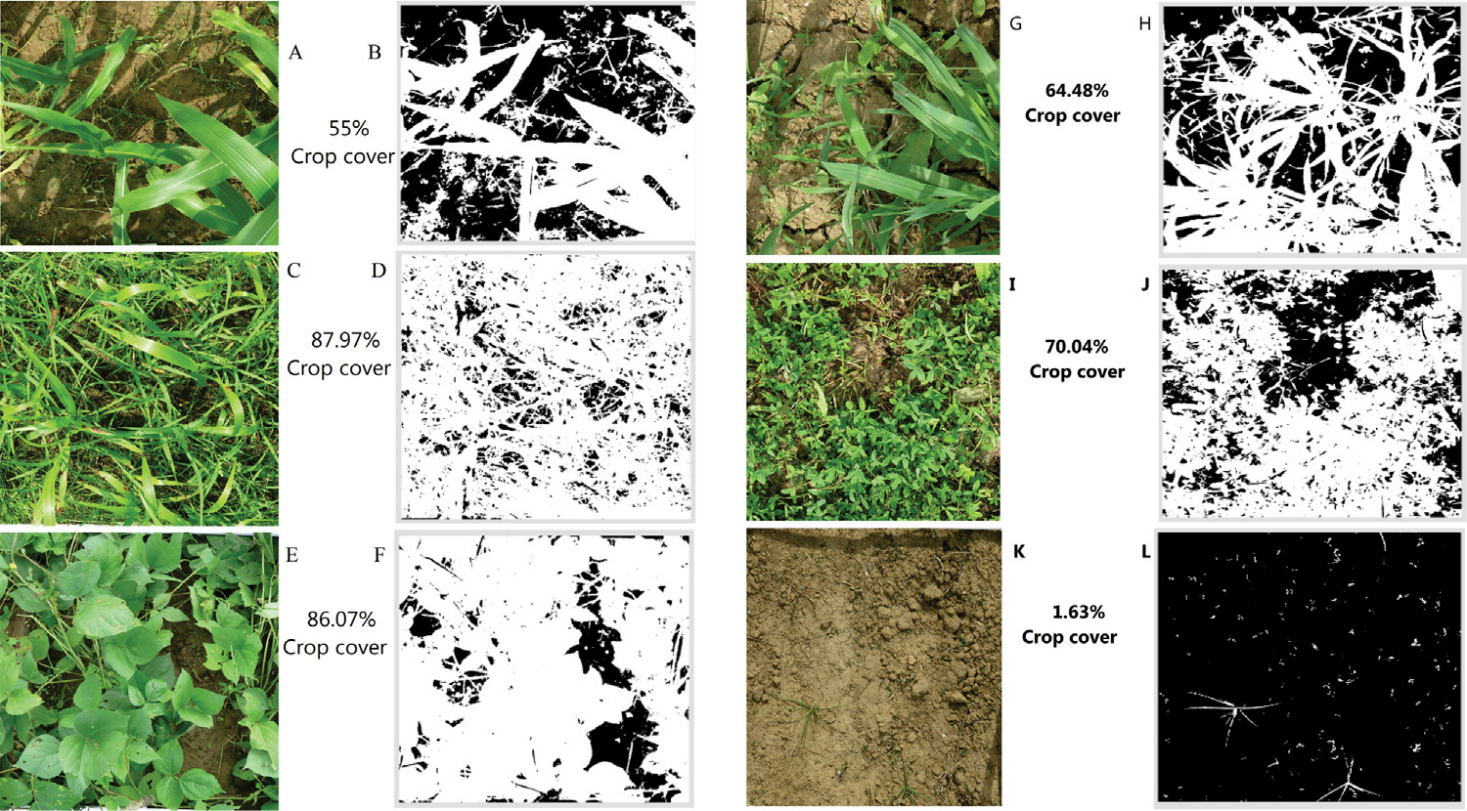
Vegetation covers estimated using Canopeo in the plots growing maize (A, B), sorghum (C, D), cowpea (E, F), oats (G, H), berseem (I, J), and bare soil (K, L) in a field study conducted at Jhansi, India. Photographs on the right (B, D, F, H, J, L) are depicting the Canopeo-converted photographs of the original photos (A, C, E, G, I, K, respectively) and white pixels are representing the green colour of the vegetation.

**Fig. 3:**
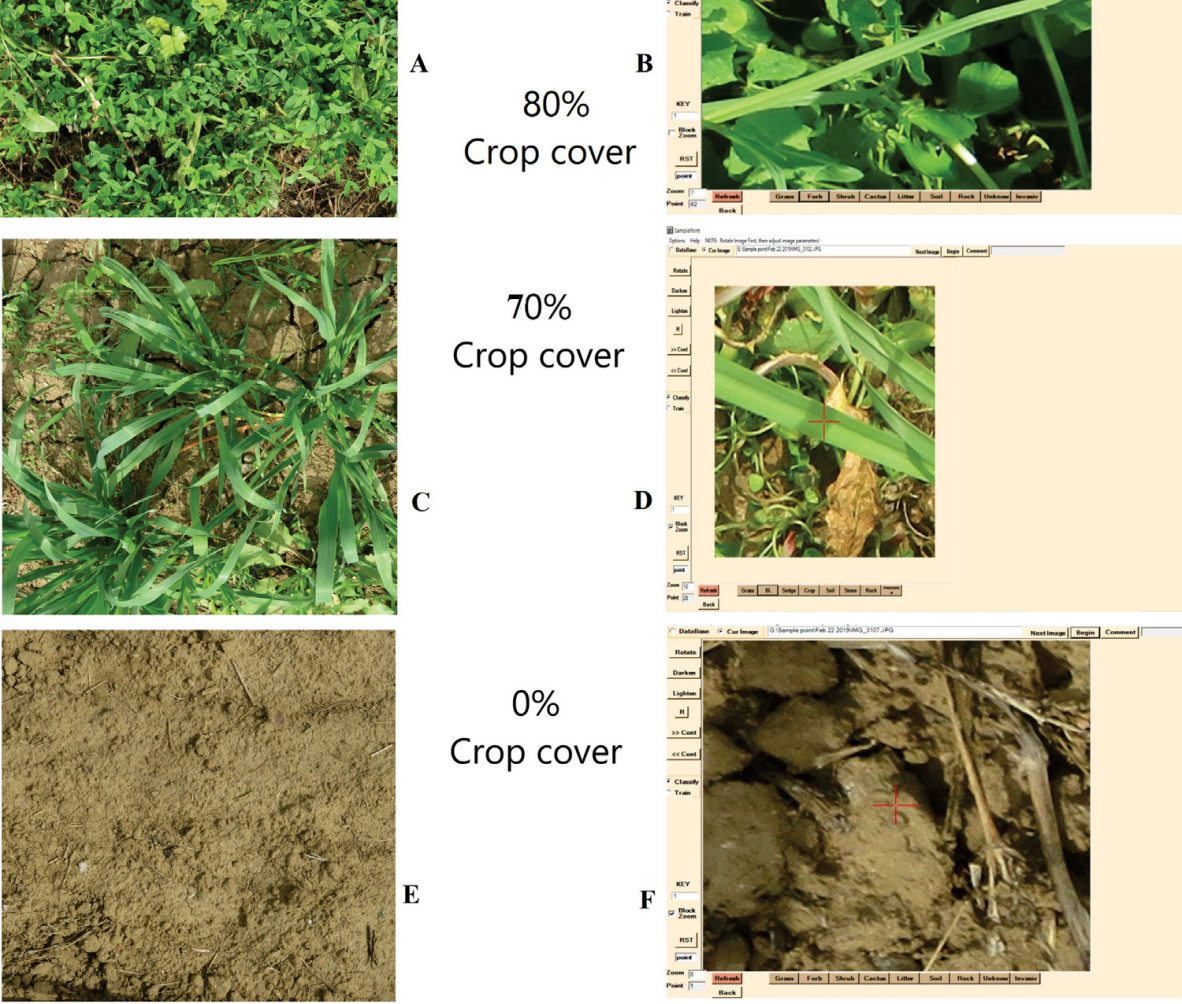
Processing of Egyptian clover (A), oats (C), and bare soil (E) images in SamplePoint (B, D and F) software.

**Fig. 4:**
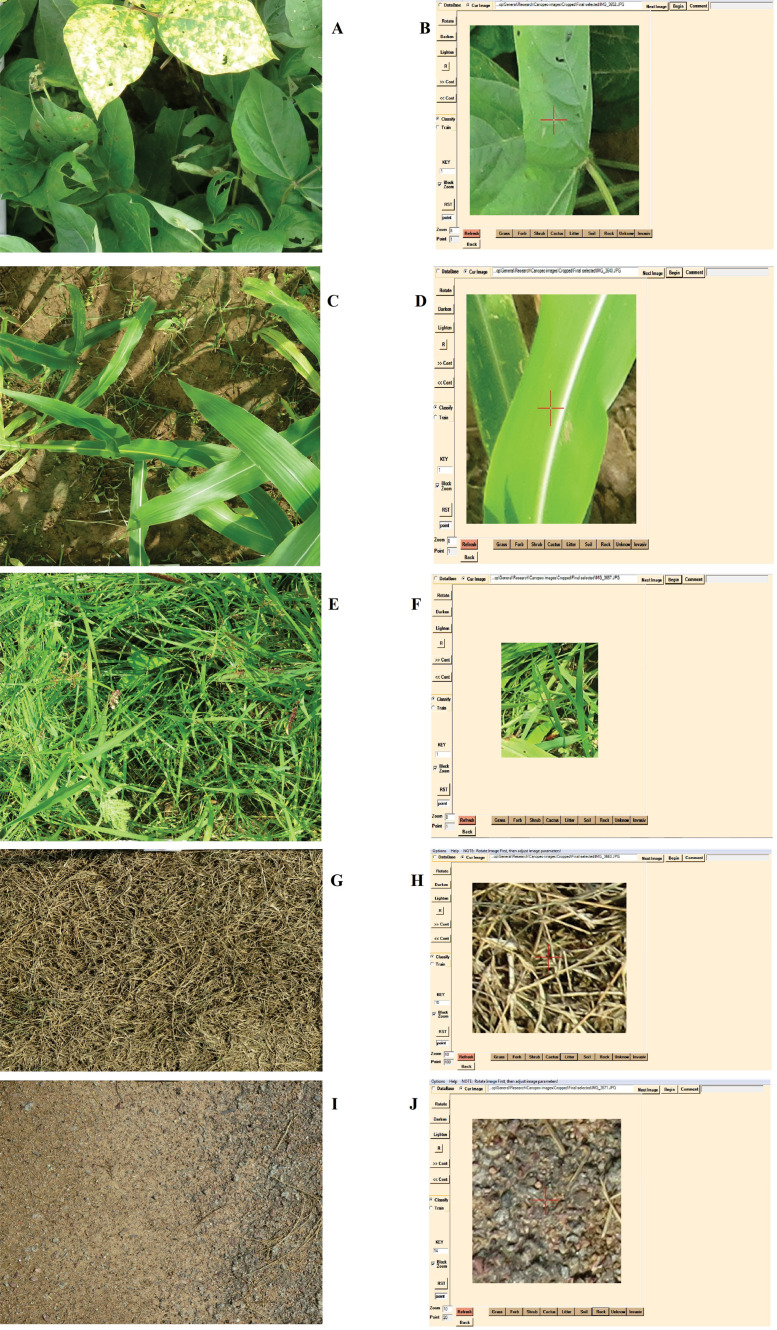
Processing of summer crops, cowpea (A), maize (C), sorghum (E), residues (G) and bare soil (I) images in SamplePoint (B, D, F, H and J) software.

**Fig. 5:**
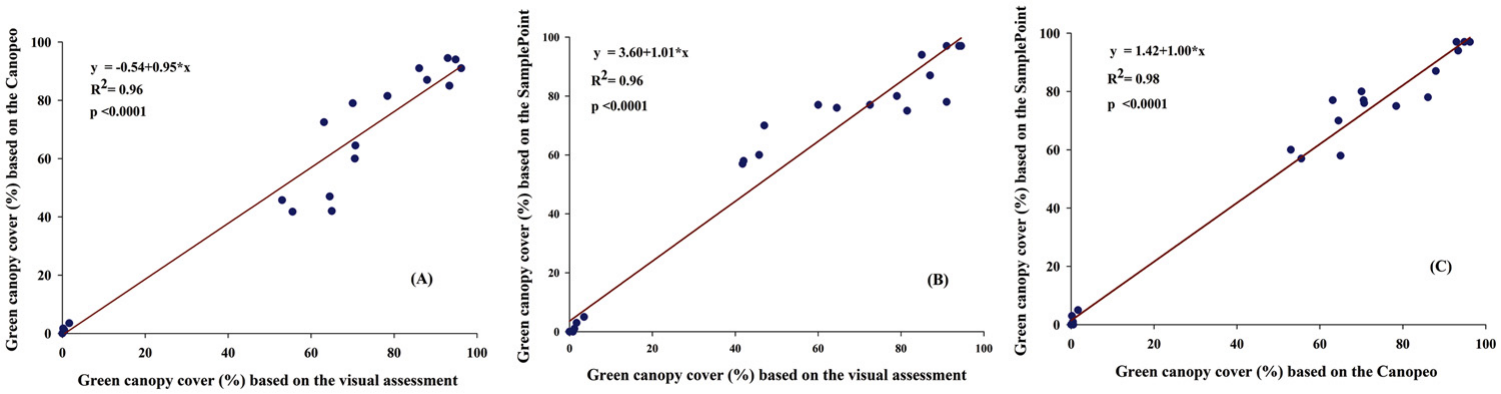
Assessments of different canopy cover measurements and their accuracy level. Relationships between (A) visual assessment and Canopeo measurement, (B) visual assessment and SamplePoint measurement, and (C) Canopeo and SamplePoint measurements are presented. Data from all the crops were pooled for the analysis.

The percentage of canopy cover (green area) based on the digital image analysis using Canopeo (Fig. 2 and 3) and SamplePoint (Fig. 4) were linearly (R^2^ = 0.96; *p* < 0.0001) related to green canopy cover percentage estimated visually (Fig. 5a and b). Therefore, it can be concluded that either of Canopeo and SamplePoint can be used for assessing the green canopy cover for forage crops. Similarly, Buchi et al. [22] reported a linear relationship between visual assessment and Canopeo data in common vetch, Egyptian clover, faba bean, field pea, hemp, mustard, oats, radish, sorghum, and sunflower (R^2^ = 0.76; *p* < 0.001) in Switzerland. However, Buchi et al. [22] found that the error associated with visual assessment was higher (11% mean absolute error) compared to Canopeo due to the underestimation of green crop cover by a human observer. Gallegos Torell and Glimskar [23] also reported the human error for estimating the crop cover using visual assessments.

Furthermore, the individual crop canopy data showed that the canopy cover estimated using Canopeo was positively correlated with the visual assessment data for cowpea (*R* = 0.94), Egyptian clover (*R* = 0.95) oats (*R* = 0.97), and sorghum (*R* = 0.51) however, it was negatively correlated for corn (*R* = - 0.61), although statically not significant (*p* > 0.05) (Table 2). The negative correlation of Canopeo with corn was possibly due to the height of corn and its larger leaf size (robustness) might have predicted more coverage of crop canopy over the visual assessment technique. In this study, corn was 85 cm tall at the time of image recording, whereas, the camera was placed at 150 cm height from the soil surface. Therefore, it is assumed that the smaller distance between the camera lens and the canopy height might have influenced the accuracy of canopy cover estimation. The crop cover estimation using SamplePoint correlated positively with visual assessment data for corn (*R* = 0.96), cowpea (*R* = 0.97), oats (*R* = 0.92), and sorghum (*R* = 0.46), whereas, the correlation coefficient (R) was negative (*R* = - 0.15) for Egyptian clover, although statically not significant (*p* > 0.05) (Table 2). Egyptian clover is an annual crop with multi-harvesting nature and the image was recorded after its first cutting, so, it is believed that the sluggish regrowth of Egyptian clover and the dead residues from the first cutting influenced the crop cover estimation by SamplePoint. In a study conducted in Switzerland, Buchi et al. [22] reported a positive correlation between visual assessment and SamplePoint in sorghum (*R* = 0.66), black oats (0.73), field pea (0.73) and Egyptian clover (0.87). The results indicated that the morphological characteristics of different plants can influence the green canopy cover estimation using different methods. Moreover, Vanha-Majamaa et al. [24] noted that the mixture of different plant populations (crop and weeds) might also affect the precision of canopy cover estimation using digital images.

Canopy cover assessed by Canopeo had a linear relationship (R^2^ = 0.98; *p* < 0.0001) with SamplePoint assessment (Fig. 5c). In a study conducted in Oklahoma, USA, Patrignani and Ochsner [5] reported that the fractional green canopy cover (FGCC) measured using Canopeo and SamplePoint were similar in corn [root mean squared difference (RMSD) = 0.074], forage sorghum (RMSD = 0.056), turf (RMSD = 0.092), and switchgrass (RMSD = 0.123). However, in our study the positive relationships between Canopeo and SamplePoint were not consistent across all the crops; the positive relationship was observed for sorghum (*R* = 0.99), cowpea (*R* = 0.97), and oats (*R* = 0.98), however, negative relationship between these two assessment methods was observed for corn (*R* = - 0.38) and Egyptian clover (*R* = - 0.44) (Table 2). Additionally, Patrignani and Ochsner [5] noted that the results could vary due to the change in plant characteristics, image quality, and weather. Buchi et al. [22] also reported that the canopy cover estimations were largely influenced by the image quality and growth stage of the crops at the time of image recording. Therefore, the canopy cover assessment using Canopeo and SamplePoint may differ from the crops, stages, image quality, and other environmental conditions.

## Conclusion

Canopy cover assessment is a measurement that can help in predicting crop productivity; however, the selection of suitable methods is necessary for precise estimation. The results of this study showed that the relationships between any two of the three assessment methods (visual assessment, Canopeo, and SamplePoint) were linear. Though the precision of Canopeo and SamplePoint may vary with several factors, this study identified that the data obtained from these two software were positively correlated for cowpea, corn, and oats. However, for crops like corn and berseem (negative correlation) selecting an appropriate stage (i.e., Knee-high stage for corn and early vegetative stage for berseem) could play a vital role in the successful use of photogrammetry software. Major limitations of these phtotgrammetry software are (1) time consumption because it is a three step procees (image capture, image processing and analysis in software), (2) influenced by crop height and dead residues around the crops, and (3) need expertise in the use of software. Our recommendation are (1) select an appropriate crop height according to the height of the camera stand (atleast 1-1.5 meter distance between the canopy and camera), (2) Select an appropriate crop stage, particularly for multicut fodder crops (choose the stage before the first cut), (3) use at least a 20 megapixel for high resolution. Overall, precise estimation of canopy cover using photogrammetry software such as Canopeo and SamplePoint can help in yield prediction, crop quality assessment, and phenotyping research.

## Declaration of Competing Interest

**A**uthors declare that they have no conflict of interest

## Data Availability

The authors do not have permission to share data.
